# The impact of norepinephrine dose reporting heterogeneity on mortality prediction in septic shock patients

**DOI:** 10.1186/s13054-024-05011-0

**Published:** 2024-07-03

**Authors:** Sebastian Morales, Pedro D. Wendel-Garcia, Miguel Ibarra-Estrada, Christian Jung, Ricardo Castro, Jaime Retamal, Luis I. Cortínez, Nicolás Severino, Greta Emilia Kiavialaitis, Gustavo Ospina-Tascón, Jan Bakker, Glenn Hernández, Eduardo Kattan

**Affiliations:** 1 Departamento de Medicina Intensiva, Facultad de Medicina, Pontificia Universidad Católica de Chile, Avenida Diagonal Paraguay 362, Santiago, Chile; 2 Institute of Intensive Care Medicine, University Hospital Zurich, University of Zurich, Zurich, Switzerland; 3 Unidad de Terapia Intensiva, Hospital Civil Fray Antonio Alcalde, Universidad de Guadalajara, Guadalajara, Jalisco Mexico; 4 The Latin American Intensive Care Network (LIVEN), https://www.livenresearch.com/; 5 Department of Cardiology, Pulmonology and Vascular Medicine, Medical Faculty, Heinrich-Heine-University Duesseldorf, Duesseldorf, Germany; 6 Cardiovascular Research Institute Düsseldorf (CARID), Heinrich-Heine University Düsseldorf, Duesseldorf, Germany; 7 División de Anestesiología, Escuela de Medicina, Pontificia Universidad Católica de Chile, Santiago de Chile, Chile; 8 Institute of Anesthesiology, University Hospital Zurich, Zurich, Switzerland; 9 Department of Intensive Care Medicine, Fundación Valle del Lili, Cali, Colombia; 10 Translational Research Laboratory in Critical Care Medicine (TransLab-CCM), Universidad Icesi, Cali, Colombia; 11 Department of Intensive Care, Erasmus MC University Medical Center, Rotterdam, Netherlands; 12 Division of Pulmonary, Allergy, and Critical Care Medicine, Columbia University Medical Center, New York, USA

**Keywords:** Norepinephrine, Norepinephrine formulation, Mortality prediction, Septic shock

## Abstract

**Background:**

Norepinephrine (NE) is a cornerstone drug in the management of septic shock, with its dose being used clinically as a marker of disease severity and as mortality predictor. However, variations in NE dose reporting either as salt formulations or base molecule may lead to misinterpretation of mortality risks and hinder the process of care.

**Methods:**

We conducted a retrospective analysis of the MIMIC-IV database to assess the impact of NE dose reporting heterogeneity on mortality prediction in a cohort of septic shock patients. NE doses were converted from the base molecule to equivalent salt doses, and their ability to predict 28-day mortality at common severity dose cut-offs was compared.

**Results:**

4086 eligible patients with septic shock were identified, with a median age of 68 [57–78] years, an admission SOFA score of 7 [6–10], and lactate at diagnosis of 3.2 [2.4–5.1] mmol/L. Median peak NE dose at day 1 was 0.24 [0.12–0.42] μg/kg/min, with a 28-day mortality of 39.3%. The NE dose showed significant heterogeneity in mortality prediction depending on which formulation was reported, with doses reported as bitartrate and tartrate presenting 65 (95% CI 79–43)% and 67 (95% CI 80–47)% lower ORs than base molecule, respectively. This divergence in prediction widened at increasing NE doses. When using a 1 μg/kg/min threshold, predicted mortality was 54 (95% CI 52–56)% and 83 (95% CI 80–87)% for tartrate formulation and base molecule, respectively.

**Conclusions:**

Heterogeneous reporting of NE doses significantly affects mortality prediction in septic shock. Standardizing NE dose reporting as base molecule could enhance risk stratification and improve processes of care. These findings underscore the importance of consistent NE dose reporting practices in critical care settings.

**Supplementary Information:**

The online version contains supplementary material available at 10.1186/s13054-024-05011-0.

## Introduction

Norepinephrine (NE) is a ubiquitously used vasoactive drug in the intensive care unit (ICU) and is considered a first line vasopressor in the management of septic shock [1–3]. The NE dose has traditionally been used as a clinical proxy for circulatory dysfunction and overall disease severity [4], and as such, it has been included in prognostic scores (i.e., SOFA score) [5]. It has also been used to define refractory shock [6] and as a trigger for additional therapies (i.e., vasopressin or corticosteroids) [7]. NE dose, either at diagnosis or its peak, has also been associated with mortality in a dose-dependent fashion [8]. Thus, it has become one of the most relevant bedside compasses for clinical decision-making.

Several salt formulations of NE (tartrate, bitartrate, or hydrochloride) are available in worldwide markets, with the preparation used varying between and even within countries [9]. Each salt has unique conversion rates to norepinephrine base, the drug's active molecule [10, 11]. A recent expert consensus raised awareness of the heterogeneity of NE dose reporting, as clinicians and researchers often fail to specify whether the administered doses are reported as salt or as its base molecule equivalent [10]. In fact, two large surveys showed that around 50% of respondents are unaware of on which formulation is NE reported locally and, therefore, administered in their practice [12], leading to potential guideline interpretation disagreements between practitioners [13]. This variability could hinder the correct application of time-sensitive clinical interventions, the comparison of results between centers, and multicentric research initiatives [2, 4, 11, 13, 14].

A crucial aspect of this reporting heterogeneity is its effect on the prognostic capacity of NE dosing. Clinicians frequently use NE dose as a risk assessment tool, often relying on specific dose cut-offs. For instance, a dose of more than 1.0 µg/kg/min has been associated with very high mortality [6, 15]. Extrapolating these thresholds from literature to clinical practice without proper standardization of dose reporting could introduce bias in mortality prediction and induce communication errors (between healthcare providers and with family members), eventually impairing the overall care process.

This study aimed to assess the impact of the heterogeneity of NE dose reporting on risk prediction in septic shock patients. We hypothesized that reporting NE dose either as base molecule or salts would generate considerable variations in mortality risk assessment. To test this hypothesis, we analyzed a cohort of septic shock patients from the MIMIC-IV database, in which NE dose was transformed from base molecule dose to equivalent doses for different salts and analyzed the predicted mortality at commonly used severity dose cut-offs.

## Methods

### Patient selection

We analyzed the data extracted from the Medical Information Mart for Intensive Care IV v2.2 (MIMIC-IV) database [16]. The MIMIC-IV database is a large open-access dataset of de-identified electronic health records from over 73,000 ICU admissions to the Beth Israel Deaconess Medical Center in Boston, Massachusetts, between 2008 and 2019. Data access was granted through physionet.org website after completion of online training on health records security. Due to the de-identified nature of data, informed consent requirement was waived.

Adult patients (≥ 18 years) who developed septic shock during their ICU stay—as defined by the Sepsis-3 criteria—were eligible [17]. Methods used to select septic shock patients are described at length in Additional File 1. In brief, this required a combination of suspicion of infection (acquisition of cultures and the start of antibiotic therapy), a SOFA score ≥ 2, norepinephrine use, and hyperlactatemia (≥ 2 mmol/L) at vasopressor initiation. Exclusion criteria were: (1) NE start 12 h before or 72 h after sepsis diagnosis, (2) missing key demographic data, and (3) absence of norepinephrine dose values after excluding outliers.

### Data extraction and study variables

Variables were extracted using a Structured Query Language (SQL) with PostgreSQL database system version 14.6 (PostgreSQL Global Development Group) and R project for statistical computing version 4.4.0 (R Foundation, Vienna, Austria). MIMIC-IV derived tables based on the centralized MIMIC code repository were used to extract relevant variables for study purposes.

NE peak dose was defined as the highest dose administered during the first 24 h after septic shock diagnosis. To avoid registry errors and spurious pump manipulations, the maximum dose had to be stable (within a 10% range) for at least 5 min to be considered as the peak dose. If not, the second highest dose was retrieved, and successive iterations were performed until these criteria were met. NE diagnosis dose was defined as the dose started or titrated closest to the septic shock diagnosis. The updated formulas published by Kotani et al. were utilized for conversion to NE equivalent score [18].

In the MIMIC-IV database, NE dose data was reported as base equivalents. These doses were converted to salt formulation doses using previously reported conversion rates, namely, multiplying by 2 for tartrate, by 1.89 for bitartrate, and by 1.22 for hydrochloride [10]. A graphical depiction of different NE dose reporting strategies and conversions to base equivalents are shown in Additional File 2 [19].

### Outcomes and statistical analysis

The primary outcome of this study was 28-day mortality. Data normality was visually assessed and tested with the Kolmogorov–Smirnov test. Descriptive statistics included counts (proportion) and medians [interquartile range]. Mixed-effects logistic regression with 95% confidence intervals was performed to predict 28-day mortality, using both the norepinephrine dose at diagnosis and peak dose as predictive variables, an interaction term for the specific salt formulation and a random effects term for the patient. Unadjusted ORs where calculated for each logistic regression and presented with its 95% confidence interval (CI). Dose distributions were analyzed through frequency histograms with Kernel density smoothing for better graphical depiction. We identified commonly used thresholds (0.1, 0.25, and 1 µg/kg/min) of norepinephrine dose to compare predicted mortality according to the dose reporting method used [1, 2, 15]. A two-tailed *p* value < 0.05 was considered significant. All calculations were performed with either GraphPad Prism v10.0 (La Joya, California, USA) or R project for statistical computing version 4.4.0 (R Foundation, Vienna, Austria).

## Results

**Fig. 1:**
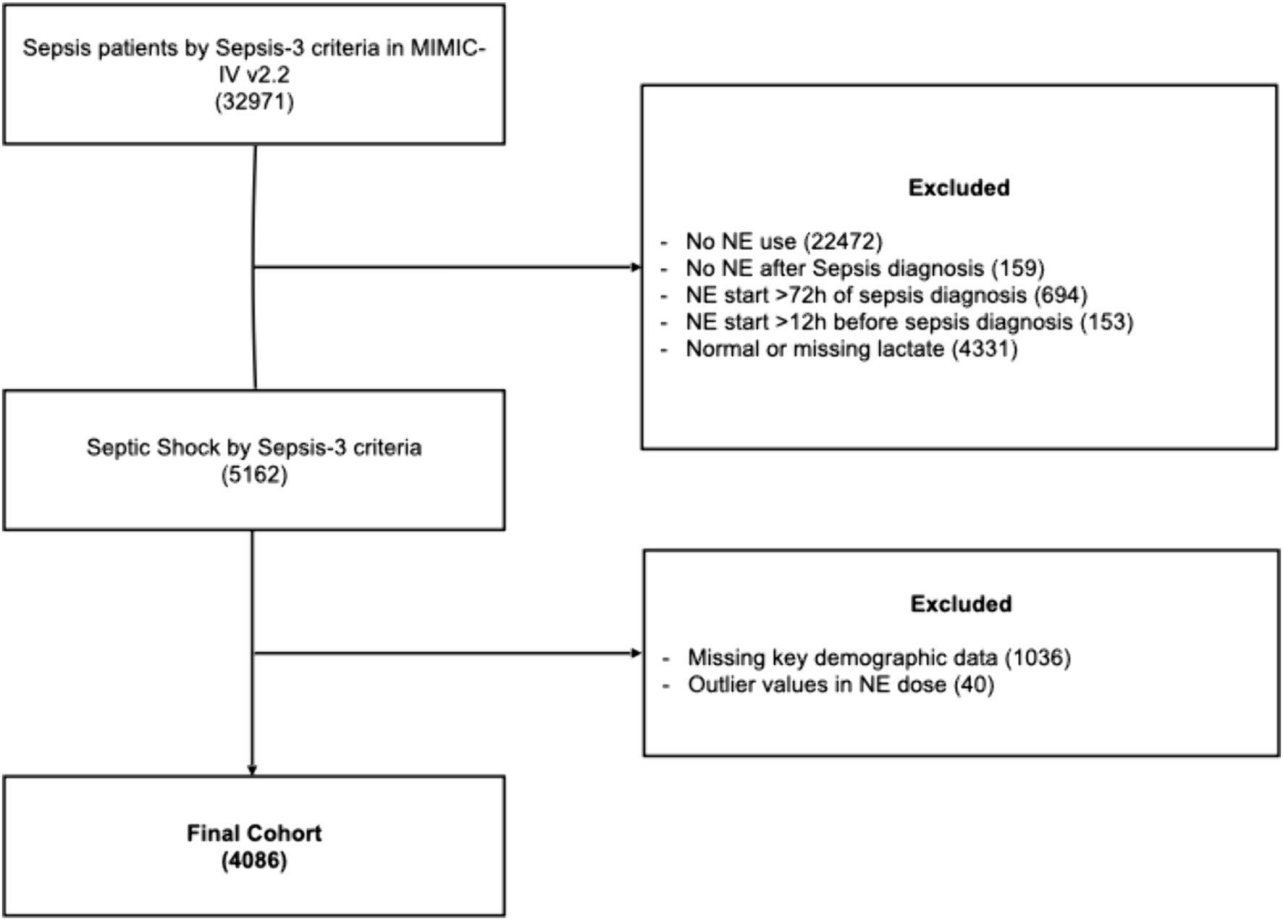
Study flow chart. Flow diagram for patient selection and cohort conformation

We identified 4086 patients fulfilling eligibility criteria and having a full dataset. A flowchart of patient selection is shown in Fig. 1. The median age was 68 [57–78] years, patients had an admission SOFA score of 7 [6–10], and a Charlson comorbidity index of 6 [4–8], as shown in Table 1. Lactate levels at diagnosis were 3.2 [2.4–5.1] mmol/L and peak NE dose during the first day was 0.24 [0.12–0.42] µg/kg/min. Regarding additional vasoactive drugs use, 2312 patients (57%) received a second vasopressor during the first 24 h, which resulted in a NE equivalent score of 0.30 [0.15–0.51]. Moreover, 3422 (84%) were on invasive mechanical ventilation and 875 (21%) required renal replacement therapy. ICU and 28-day mortality was 32.8% and 39.3%, respectively.

**Table 1:** Key demographic variables, severity scoring, life support requirements, and outcomes of the study cohort

Variable	Value
Age (years), median [IQR]	68 [57–78]
Female, No. (%)	1662 (40.7)
Weight (kg), median [IQR]	79 [67–94]
BMI (kg/m^2^), median [IQR]	27.7 [23.8–32.3]
Charlson Comorbidity Index, median [IQR]	6 [4–8]
SAPS II, median [IQR]	50 [40–61]
SOFA score, median [IQR]	7 [6–10]
Lactate at diagnosis (mmol/L), median [IQR]	3.2 [2.4–5.1]
Lactate peak (mmol/L), median [IQR]	3.6 [2.4–6.5]
Norepinephrine at diagnosis µg/kg/min base molecule, median [IQR]	0.1 [0.05–0.2]
Peak norepinephrine within first 24 h (µg/kg/min base molecule), median [IQR]	0.24 [0.12–0.42]
Norepinephrine as unique vasopressor, No. (%)	1774 (43.4)
Other vasopressors used, No. (%)	
*Vasopressin*	1526 (37.3)
*Epinephrine*	777 (19.0)
*Phenylephrine*	1051 (25.7)
Max NE equivalents at 24 h, median [IQR]	0.30 [0.15–0.51]
Fluid boluses administered in the 24 h before diagnosis (ml), median [IQR]	1000 [1000–2000]
Fluid boluses administered in the 24 h after diagnosis (ml), median [IQR]	1000 [1000–3500]
Invasive Mechanical Ventilation, No. (%)	3422 (84)
Renal Replacement Therapy, No. (%)	875 (21.4)
ICU LOS (days), median [IQR]	4.8 [2.5–9.7]
Hospital LOS (days), median [IQR]	11.0 [6.0–21.0]
ICU mortality, No. (%)	1341 (32.8)
28-day mortality, No. (%)	1607 (39.3)

BMI, body mass index; SAPS, simplified acute physiological score, SOFA, sequential organ failure assessment; NE, norepinephrine; ICU, intensive care unit; LOS, length of stay

Additional File 2 shows the linear divergence between different reporting strategies and base molecule conversion when compared at the same reported dose. The frequency distribution of the NE dose at diagnosis and peak NE dose reported as base molecule and their conversions to doses as salt formulations are shown in Additional File 3.

**Fig. 2:**
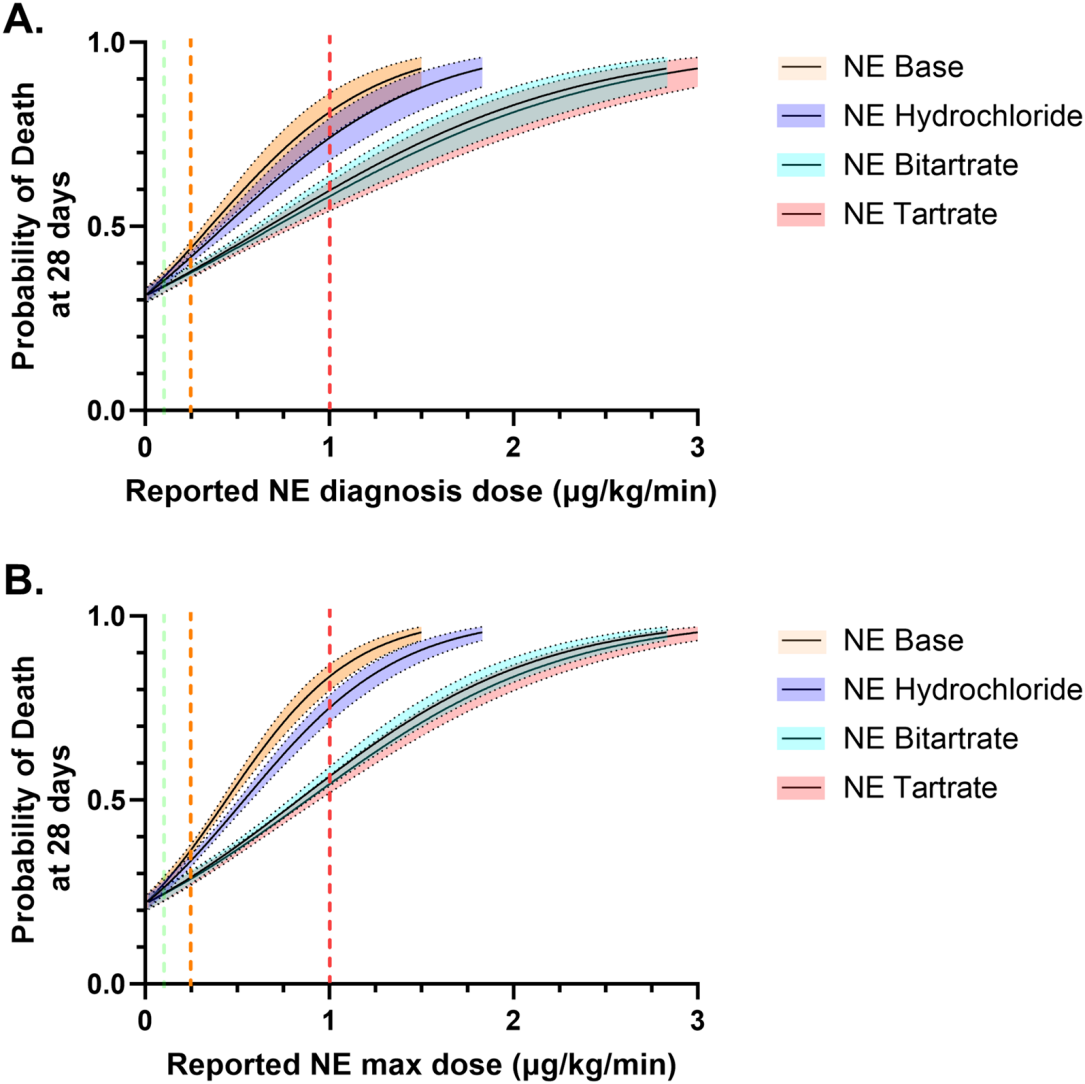
28-day mortality prediction according to norepinephrine dose reporting method. Panel A represents NE diagnosis dose, while panel B represents maximum dose during the first 24 h since diagnosis. Dashed lines represent different commonly used thresholds of norepinephrine dose: green line marks 0.1 µg/kg/min (defining the highest value for cardiovascular domain in the SOFA score), orange 0.25 µg/kg/min (suggested dose to initiate vasopressin and/or hydrocortisone) and red 1 µg/kg/min (widely considered as “high-dose” vasopressor)

Dose-response analyses of the base NE dose at diagnosis for 28-day mortality revealed an odds ratio of 9.4 (95% CI 6.1–14.7) (Fig. 2A). By contrast, the hydrochloride formulation presented a 33 (95% CI − 18–62, *p* = 0.16)% lower odds ratios than the base formulation, whereas the bitartrate and tartrate formulations presented 65 (95% CI 43–79, *p* < 0.001)% and 67 (95% CI 47–80, *p* < 0.001)% lower odds ratios, respectively. The dose–response analyses for the maximal norepinephrine dose within 24 h from diagnosis had a similar behaviour (Fig. 2B). The ORs for both curves are shown in Additional File 4. Additional File 5 depicts a similar curve when dose–response analysis is plotted for peak NE equivalent dose.

When comparing the most divergent formulations (base and tartrate)—illustrated in Table 2—at different pre-defined cut-off values, the variability of risk prediction increased considerably by increasing NE dose. As shown in Fig. 2B, at 0.1 µg/kg/min reported dose, the absolute difference in the predicted mortality between NE base and tartrate was of 3% (27 [25–29] vs. 24% [22–27], respectively). In contrast, at 1 µg/kg/min reported dose, the absolute difference was 29% (83% [80–87] vs. 54% [52–56], respectively). Table 2 shows a detailed comparison of these results.

**Table 2:** Probability of death of commonly used cut-off values of norepinephrine dose according to the reported formulation

Dose (µg/kg/min)	Base % (95% CI)	Hydrochloride % (95% CI)	Bitartrate % (95% CI)	Tartrate % (95% CI)
*NE dose at diagnosis*				
0.1	36 (34–38)	35 (34–37)	34 (32–36)	33 (32–35)
0.25	44 (42–46)	42 (40–43)	38 (36–39)	37 (36–39)
1.0	81 (74–86)	74 (68–79)	60 (56–64)	58 (54–62)
*NE peak dose during the first 24 h*				
0.1	27 (25–29)	26 (24–28)	25 (23–27)	24 (22–27)
0.25	36 (34–38)	34 (32–35)	29 (27–31)	28 (26–30)
1.0	83 (80–87)	75 (71–79)	57 (54–59)	54 (52–56)

OR, odds ratio; NE, norepinephrine; CI, confidence interval

## Discussion

The results of this study can be summarized as follows: the same reported dose of norepinephrine, depending on whether salt formulations or the base molecule equivalence is used, may lead to relevant heterogeneity in mortality prediction, and this divergence progressively increases as a function of the dose itself.

Even though previous studies have assessed the heterogeneity of mortality risk relative to the reported norepinephrine formulation, they compared patient populations with diverse baseline characteristics, disease severity, and were subjected to different care processes, thus adding potential confounders to the comparison, and limiting conclusions [20]. Conversely, as our study compares the same population under the optics of different NE dose reporting methods, the factors that could influence mortality risk are inherently controlled. This allowed us to perform a continuous assessment of mortality prediction [8], contrasting the behavior of the reported drug dose by formulation in a dose-dependent fashion and accurately quantifying its impact and trajectory, rather than comparing only point estimates.

Since the association between NE dose and mortality corresponds to a sigmoid function curve, the predicted mortality gap between different norepinephrine formulations increases significantly at higher doses. This phenomenon is relevant since it can amplify cognitive biases into clinical risk prediction. Thus, if half of the clinicians are unaware of how norepinephrine dose is reported in their clinical practice [12] and how severity cut-offs were constructed, the latter are liable to be misinterpreted. In international surveys, the most frequently used formulation was tartrate salt, which, as was shown in this study, has the highest divergence on risk prediction if reported as such compared to the base molecule dosing strategy [4, 12, 13]. This could hinder the clinical decision-making process, timely installment of therapeutic interventions, and effective communication among relevant stakeholders, potentially delivering suboptimal clinical care, as stressed by Salvati et al. [13].

This study has several limitations. First, those inherent to database analyses, including coding errors, outliers, or missing data on key variables (such as withdrawal or withholding care). The database was derived from a single hospital throughout a prolonged timespan, in which care evolved in the light of scientific progress. Also, even though we focused on norepinephrine dose as a proxy for severity, other vasoactive drugs were used in a considerable proportion of patients. However, when other drugs were transformed into NE equivalents, the main driver (almost 75%) of the equivalent dose was norepinephrine, confirming it was the hegemonic drug in the cohort.

In conclusion, norepinephrine dose reporting, depending on the formulation used, leads to considerable heterogeneity in mortality prediction in septic shock patients, with increasing divergences at higher-end doses. Efforts to prospectively standardize NE drug reporting as base molecule by the international clinical community should be sought to homogenize the process of care, risk stratification, and improve patient-centered outcomes.

### Supplementary Information

Supplementary Material 1: Definitions for “sepsis” and “septic shock” from data available on MIMIC-IV database.

Supplementary Material 2: Norepinephrine base molecule equivalent dose for increasing doses reported as salt formulations. Dashed lines represent different commonly used thresholds of norepinephrine dose: green line marks 0.1 µg/kg/min (defining the highest value for cardiovascular domain in the SOFA score), orange 0.25 µg/kg/min (suggested dose to initiate vasopressin and/or hydrocortisone and red 1 µg/kg/min (widely considered as “high-dose” vasopressor). Adapted from reference 19.

Supplementary Material 3: Distribution of diagnosis NE dose and peak NE dose, including corresponding conversions to doses reported as salts.

Supplementary Material 4: Odds Ratio (95% CI) of the NE dose for each salt or base molecule to predict 28-day mortality.

Supplementary Material 5: 28-day mortality prediction according to peak norepinephrine equivalents at 24 h.

## Data Availability

All the raw data used in this manuscript is publicly available for accredited researchers. The dataset used in this study, MIMIC-IV, is available at https://mimic.physionet.org. All code used for extracting, cleaning, filtering and modelling will be made available in the future, ensuring end-to-end reproducibility of all results presented. Nevertheless, it is available from the corresponding author on reasonable request.

## Abbreviations

NE: Norepinephrine
OR: Odds ratio
SOFA: Sequential organ failure assessment
MIMIC-IV: Medical Information Mart for Intensive Care IV v2.2
BMI: Body mass index
SAPS II: Simplified Acute Physiology Score II
ICU: Intensive care unit
LOS: Length of stay
