# Supplementary material for: The impact of norepinephrine dose reporting heterogeneity on mortality prediction in septic shock patients
Source: Crit Care. 2024 Jul 3;28:216. doi: 10.1186/s13054-024-05011-0 (PMC11220947; doi:10.1186/s13054-024-05011-0)
Supplement: Supplementary file 1 — Supplementary Material 1: Definitions for “sepsis” and “septic shock” from data available on MIMIC-IV database. [file 13054_2024_5011_MOESM1_ESM.docx]

1. Definition of “Sepsis”

A notable limitation of the MIMIC-IV database is the absence of the documentation of diagnoses made by physicians during clinical practice. Instead, it only includes ICD-9 and ICD-10 codes, which are assigned by trained personnel who review signed clinical notes post-discharge without a specific timeframe for the diagnosis^1^. For this study, sepsis was defined according to previously established methods^2,3^, aiming to align with the Third International Consensus Definitions for Sepsis (Sepsis-3), which emphasize clinical suspicion of infection and the presence of end-organ dysfunction^4^. The diagnosis of sepsis was computed using three criteria: (1) suspected infection, identified by the earlier timestamp of antibiotic administration and blood culture sampling within a defined timespan. Specifically, if antibiotics were administered first, cultures had to be obtained within 24 hours; conversely, if cultures were obtained first, antibiotics had to be administered within 72 hours. (2) Given the limitation of lacking baseline patient characteristics prior to ICU admission, end-organ dysfunction was defined as a SOFA score of two or more points, with the implicit assumption that the baseline SOFA score was zero before ICU admission. (3) The onset of sepsis was defined as the earliest time at which a patient exhibited a SOFA score ≥ 2 (t_SOFA_) and suspicion of infection (t_infection_), provided that t_SOFA_ occurred no more than 48 hours before or 24 hours after t_infection_; otherwise, the patient was not classified as having sepsis^3^. The Structured Query Language (SQL) code utilized for data extraction is available at [mimic-iv/concepts/sepsis at master · MIT-LCP/mimic-iv · GitHub](https://github.com/MIT-LCP/mimic-iv/tree/master/concepts/sepsis).

1. Definition of “Septic Shock”

Patients with septic shock were identified based on three primary criteria: (1) a diagnosis of sepsis (as defined above), (2) the initiation of norepinephrine infusion within a specific timeframe, and (3) the presence of hyperlactatemia (≥2 mmol/L) at the start of vasopressor therapy. To mitigate confounders related to vasoactive drug use and organ dysfunction in the diagnosis of septic shock, patients who commenced norepinephrine infusion more than 12 hours before or more than 72 hours after the sepsis diagnosis were excluded. Given the limitations of laboratory measurement registries and to allow for flexibility in patient classification, a timeframe of 12 hours before and 12 hours after the initiation of norepinephrine infusion was used to identify elevated lactate levels indicative of hyperlactatemia. This specific timestamp—NE infusion start—was employed to select patients with elevated lactate levels most likely attributable to systemic hypoperfusion in the context of hemodynamic instability.

References

1. Johnson AEW, Bulgarelli L, Shen L, Gayles A, Shammout A, Horng S, et al. MIMIC-IV, a freely accessible electronic health record dataset. Sci Data. 2023 Jan 3;10(1):1.

2. Nemati S, Holder A, Razmi F, Stanley MD, Clifford GD, Buchman TG. An Interpretable Machine Learning Model for Accurate Prediction of Sepsis in the ICU. Critical Care Medicine. 2018 Apr;46(4):547–53.

3. Hu W, Chen H, Ma C, Sun Q, Yang M, Wang H, et al. Identification of indications for albumin administration in septic patients with liver cirrhosis. Crit Care. 2023 Jul 28;27(1):300. Suppl 1: 1-2.

4. Evans L, Rhodes A, Alhazzani W, Antonelli M, Coopersmith CM, French C, et al. Surviving sepsis campaign: international guidelines for management of sepsis and septic shock 2021. Intensive Care Med [Internet]. 2021; Available from: http://www.ncbi.nlm.nih.gov/pubmed/34599691
