# Supplementary figures and images for: The impact of norepinephrine dose reporting heterogeneity on mortality prediction in septic shock patients
Source: Crit Care. 2024 Jul 3;28:216. doi: 10.1186/s13054-024-05011-0 (PMC11220947; doi:10.1186/s13054-024-05011-0)

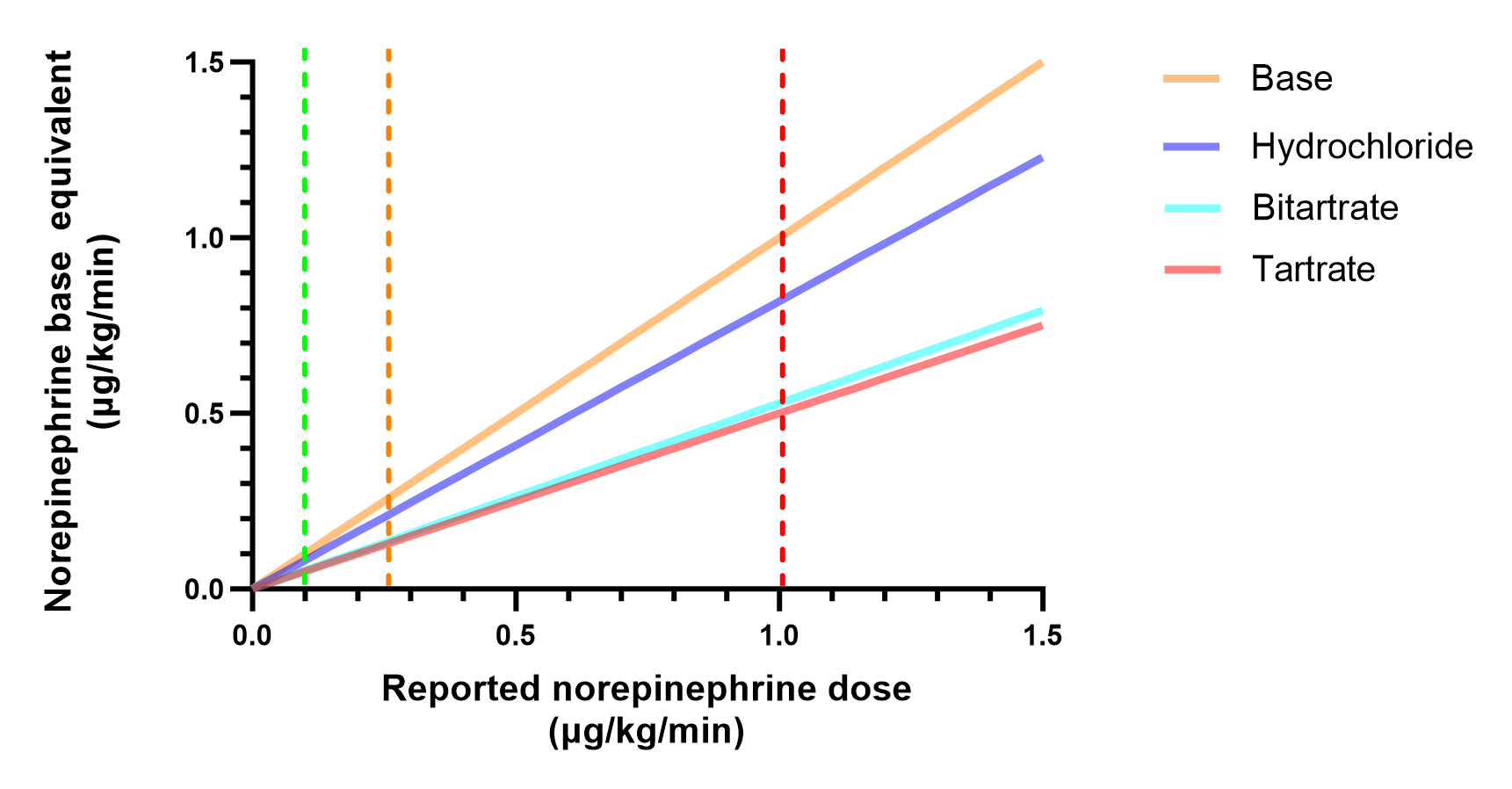

Supplement: Supplementary file 2 — Supplementary Material 2: Norepinephrine base molecule equivalent dose for increasing doses reported as salt formulations. Dashed lines represent different commonly used thresholds of norepinephrine dose: green line marks 0.1 µg/kg/min (defining the highest value for cardiovascular domain in the SOFA score), orange 0.25 µg/kg/min (suggested dose to initiate vasopressin and/or hydrocortisone and red 1 µg/kg/min (widely considered as “high-dose” vasopressor). Adapted from reference 19. [file 13054_2024_5011_MOESM2_ESM.tif]

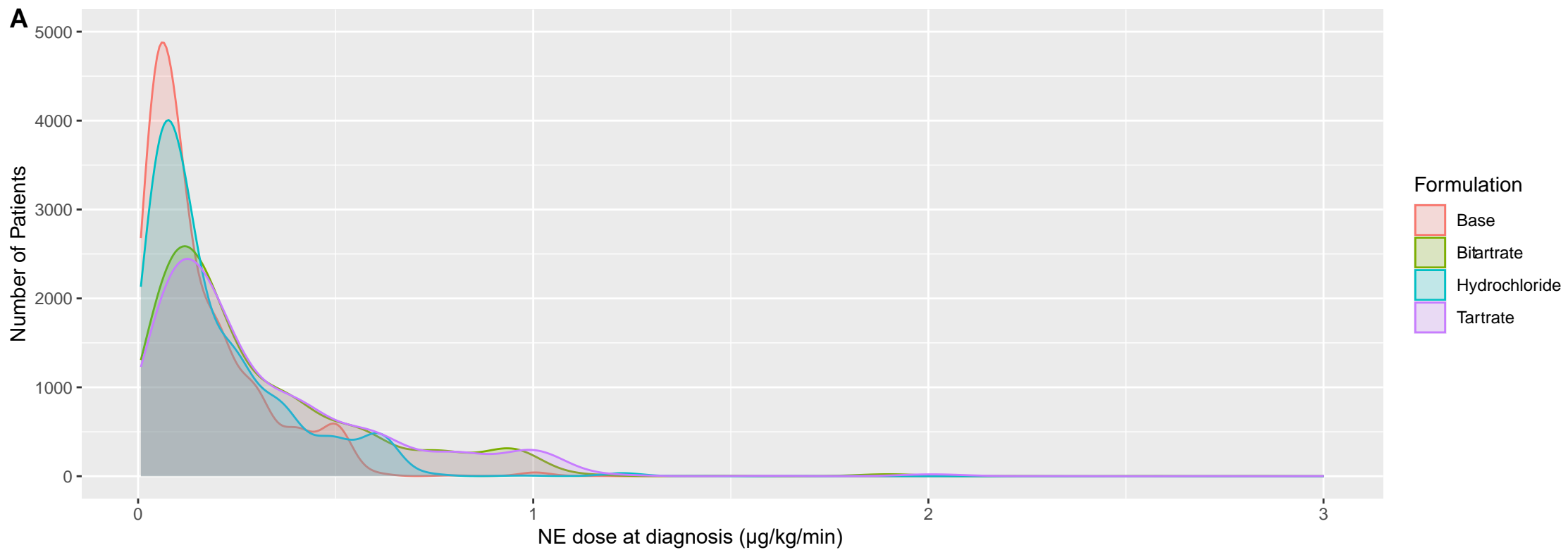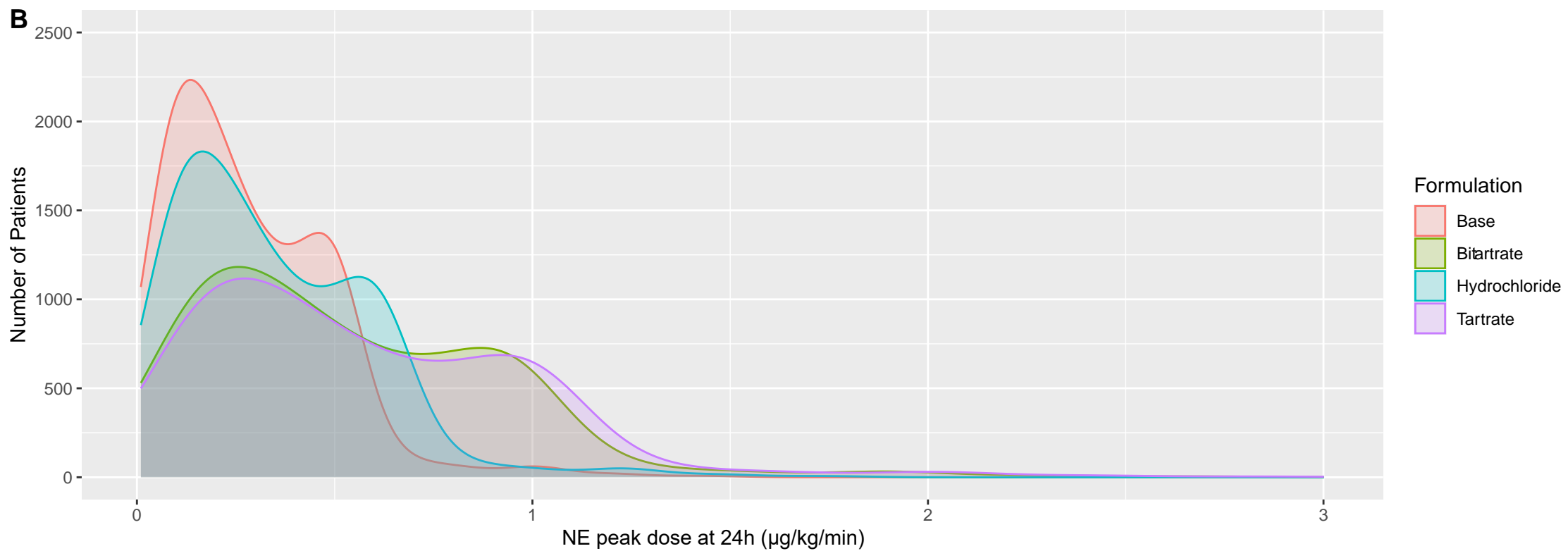

Supplement: Supplementary file 3 — Supplementary Material 3: Distribution of diagnosis NE dose and peak NE dose, including corresponding conversions to doses reported as salts. [file 13054_2024_5011_MOESM3_ESM.pdf]

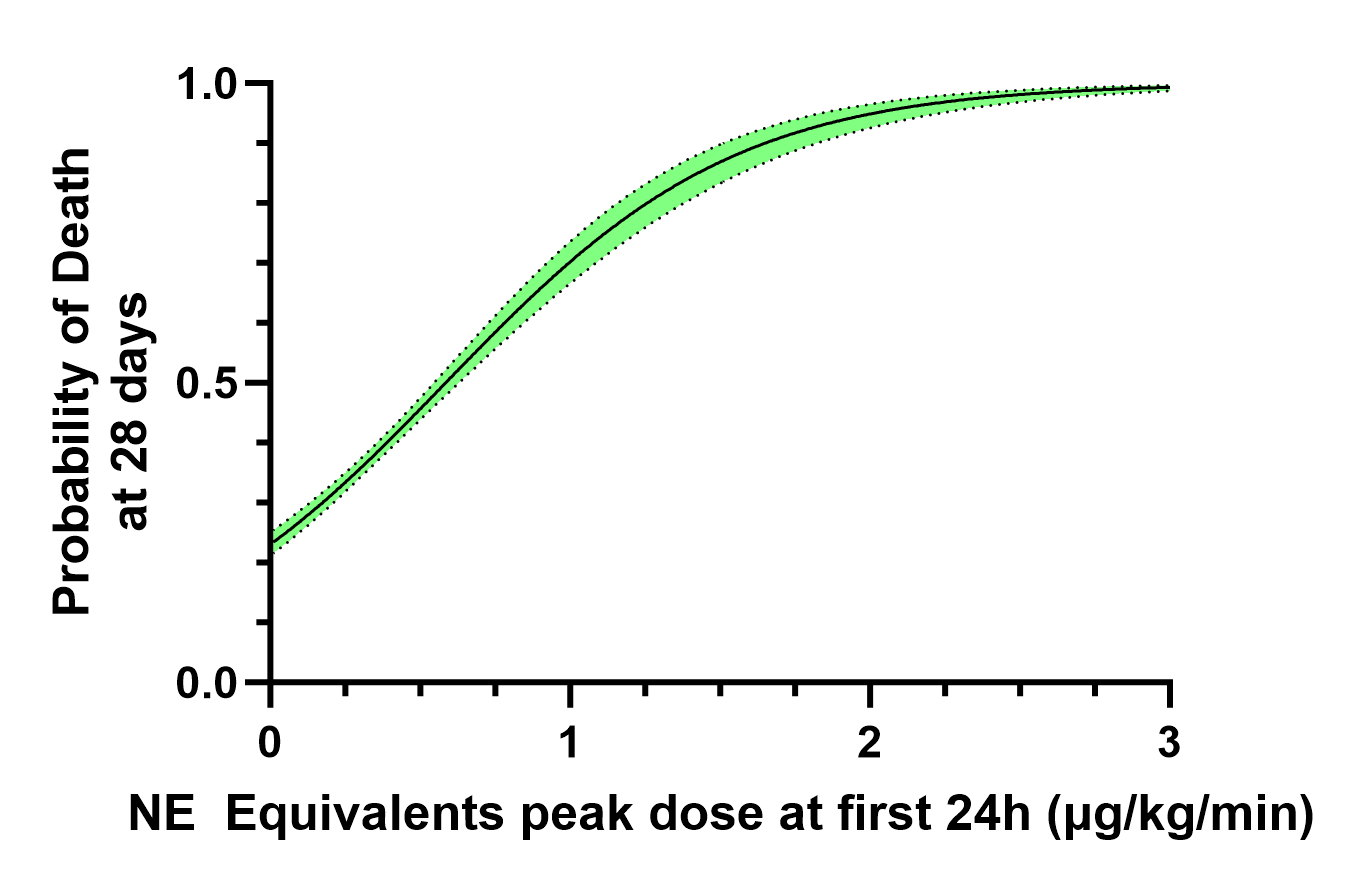

Supplement: Supplementary file 5 — Supplementary Material 5: 28-day mortality prediction according to peak norepinephrine equivalents at 24 h. [file 13054_2024_5011_MOESM5_ESM.tif]
