# Supplementary material for: The impact of norepinephrine dose reporting heterogeneity on mortality prediction in septic shock patients
Source: Crit Care. 2024 Jul 3;28:216. doi: 10.1186/s13054-024-05011-0 (PMC11220947; doi:10.1186/s13054-024-05011-0)
Supplement: Supplementary file 4 — Supplementary Material 4: Odds Ratio (95% CI) of the NE dose for each salt or base molecule to predict 28-day mortality. [file 13054_2024_5011_MOESM4_ESM.docx]

**Additional File 3**: **Odds Ratio (95% CI) of the NE dose for each salt or base molecule to predict 28-day mortality.**

|  | **Diagnosis NE Dose** | **p-value** | **Peak NE Dose** | **p-value** |
| --- | --- | --- | --- | --- |
| Base | 9.4 (6.1-14.7) |  | 18.1 (12.8-25.8) |  |
| Hydrochloride | 6.3 (4.4-9.0) | 0.162 | 10.8 (8.1-14.3) | 0.023 |
| Bitartrate | 3.3 (2.6-4.1) | <0.0001 | 4.6 (3.9-5.6) | <0.0001 |
| Tartrate | 3.1 (2.5-3.8) | <0.0001 | 4.3 (3.6-5.1) | <0.0001 |

NE: norepinephrine

ORs calculated per 1 mcg/kg/min change of NE dose.
